# The role of Anti-VEGF agents in treatment of neovascular glaucoma

**DOI:** 10.22336/rjo.2022.41

**Published:** 2022

**Authors:** Mădălina-Casiana Palfi (Salavat), Edward Paul Șeclăman, Ramona Barac, Emil Ungureanu, Gabriel Iorgu, Andrada Artamonov, Laurențiu Leuștean, Mădălina Veronica Borugă

**Affiliations:** * Department of Ophthalmology, “Victor Babeș” University of Medicine and Pharmacy, Timișoara, Romania; ** Department of Biochemistry, “Victor Babeș” University of Medicine and Pharmacy, Timișoara, Romania; *** Department of Ophthalmology, “Carol Davila” University of Medicine and Pharmacy, Bucharest, Romania; **** Clinical Emergency Eye Hospital, Bucharest, Romania; ***** Department of Toxicology and Drug Industry, “Victor Babeș” University of Medicine and Pharmacy, Timișoara, Romania

**Keywords:** neovascular glaucoma, anti-VEGF, Bevacizumab, Avastin, intravitreal injection, neovascularization

## Abstract

**Aim:** The aim of this study was to show the efficacy of intravitreal treatment with Bevacizumab (Avastin) in patients with secondary neovascular glaucoma, in different stages of the disease.

**Method:** A retrospective study was performed on 67 patients with neovascular glaucoma. The main parameters evaluated were the patients’ history, slit lamp examination, visual acuity, ocular tonometry, fundus examination, gonioscopy, and visual field.

**Results:** It was observed that the pathology had a preponderance in males of the 6th decade, with frequently unilateral damage. Patients were referred to an ophthalmologist when the diseases reached an advanced stage, usually when the visual acuity had no light perception and the intraocular pressure was over 45 mmHg. However, the treatment with Avastin intravitreal showed a good evolution, with regression of neovessels in the first 4-7 days and maintenance of intraocular pressure within normal limits in about 60% of cases, 3 months after injection.

**Conclusion:** The most effective treatment in secondary neovascular glaucoma is the correct therapy of the main disease. The association of Avastin and laser photocoagulation leads to regression in iris and retinal neovessels.

**Abbreviations:** anti-VEGF = anti-Vascular Endothelial Growth Factor, PDGF = Platelet Derived Growth Factor, bFGF = basic Fibroblast Growth Factor

## Introduction

Neovascular glaucoma represents a special pathology, which consists of the proliferation of neovessels on the surface of the iris, but also at the iridocorneal angle, in the trabecular meshwork. This condition represents the final stage of the retinal ischemic complication [**1**].

Angiogenesis occurs mainly through ischemia, but inflammation and hypoxia are also mechanisms that can lead to neovascular formation. It is known that there is a factor of angiogenesis, which by hypoxia activates the proliferation of neovessels [**2**].

Hypoxia is involved in cases associated with proliferative retinopathy. By compensation, the diffusing part of the oxygen from the aqueous humor to the posterior leads to hypoxia of the iris. This causes the secretion of proangiogenic factors, such as VEGF (vascular endothelial growth factor), PDGF (platelet derived growth factor) and bFGF (basic fibroblast growth factor) [**2**].

VEGF is the best-known proangiogenic factor and has 4 isoforms; variant A is most often involved in iris neovascularization [**2**].

The angiogenesis process begins as follows: small spaces are formed between the cells of the endothelium capillary wall, allowing the passage of proteins and fibrinogen, the latter turning into fibrin and forming a temporary matrix for the neovessel. Then, the endothelial cells are organized, forming the vascular bud, and their proliferation leads to the formation of a thin vascular lumen [**2**].

The neovessels are formed on a fibrovascular support (fibrovascular membrane), which contains inflammatory cells, macrophages, lymphocytes and collagen fibers [**2**].

There are many causes that can lead to secondary neovascular glaucoma, among which the following should be mentioned [**1**,**3**,**4**]:

• Ocular vascular disease: central retinal vein obstruction, diabetic retinopathy, central retinal artery obstruction, Coats disease, retinopathy of prematurity, etc.

• Extraocular vascular disease: Horton’s arteritis, Takayasu disease, carotid-cavernous fistula, etc.

• Other eye disease: rhegmatogenous retinal detachment, chronic uveitis, endophthalmitis, sympathetic ophthalmia, etc.

• Ocular neoplasms: melanoma, retinoblastoma, hemangioma, metastasis.

• Postoperative: cataract, retinal detachment, vitrectomy.

*Clinical features:*

• Stage I: vascular proliferation at the edge of the pupil, neovessels with thin, fine wall, difficult to observe in patients with dark iris, +/- cells and Tyndall positive in the anterior chamber, neovessels in the anterior chamber, visible at gonioscopy.

• Stage II: hyperemia and increased intraocular pressure because of fibrovascular membrane formation (that cannot be identified at gonioscopy), leading to open-angle glaucoma, uveal ectropion, ocular pain and loss of visual acuity.

• Stage III: the same modifications seen in stage II, plus synechia and progressive angle closure (by contraction of the fibrovascular membrane, with the traction of the iris over the trabecular meshwork).

The diagnosis is based on the evaluation of the medical history (diabetes, hypertension, atherosclerosis and previous history of decreased visual acuity), slit lamp evaluation and gonioscopy (there are cases without neovascularization on the pupillary margin, but with direct formation of the neovessels in the iridocorneal angle) and angiography with iris fluorescein to observe neovessels that are difficult to see in early stages [**5**].

Positive diagnosis: slit lamp examination, intraocular pressure, gonioscopy, fundus examination, and visual field [**6**].

Differential diagnosis of neovascular glaucoma: uveitic glaucoma, acute glaucoma, chronic closure of the iridocorneal angle, Iridocorneal Endothelial Syndrome, Fuchs heterochromia iridocyclitis [**1**]. In this pathology, treatment efficiency is reduced, sometimes with discouraging results, but it is necessary to be followed because in its absence, the pathology evolves towards progressive deterioration of the visual acuity, followed by atrophy of the eyeball and significant pain. The prognosis is severe [**7**-**9**]. In addition to the local treatment with antiglaucoma eye drops and surgery, intravitreal administration of Bevacizumab (Avastin), an anti-VEGF agent, showed optimistic results. Bevacizumab can inhibit the neoformation of vessels, so it is used as a treatment in neovascular glaucoma. The half-life is estimated to be 20 days, and the clearance varies depending on certain constitutional factors. The intravitreal injection may cause side effects such as uveitis, subconjunctival hemorrhage, endophthalmitis, eyelid irritation and retinal detachment [**7**-**9**].

## Materials and method

A retrospective study was performed on 67 patients, which aimed to evaluate the effectiveness of the intravitreal treatment with Bevacizumab (Avastin) on visual impairment and pain. The study concentrated on medical history, ophthalmological examination, visual acuity, tonometry, fundus examination, gonioscopy, and visual field.

The treatment was focused on lowering the intraocular pressure and maintaining visual function. Initially, an attempt was made to reduce intraocular pressure by administrating local combinations of beta blockers, prostaglandin analogues and carbonic anhydrase inhibitors, associated with systemic administration of carbonic anhydrase inhibitors. The improvement of the symptoms of the underlying disease was also considered. The treatment regimen included intravitreal injections with Bevacizumab (Avastin), in a first stage, followed by retinal pan photocoagulation.

## Results

Distribution by gender: the vast majority were males (51%). Ocular involvement: 93% of patients had monocular involvement, while 7% of patients had binocular involvement.

Regarding the distribution by age, most of the patients were between 60-69 years old (37%), 24% between 70-79 years old, 21% between 50-59 years old, 15% between 80-89 years old, and only 3% were between 40-49 years old.

The background disease was represented by diabetic retinopathy (56%), followed by center retinal vein obstruction (31%), retinal detachment (12%) and center retinal artery obstruction (1%).

Most patients had the visual acuity affected at admission: 43% no light perception, 21% hands motion, 14% light perception. Also, in 7% of cases the visual acuity was 5/ 50, another 7% of patients had the visual acuity 2/ 50, followed by 5% with a visual acuity 1/ 50, and 3% with 3/ 50.

**Fig. 1:**
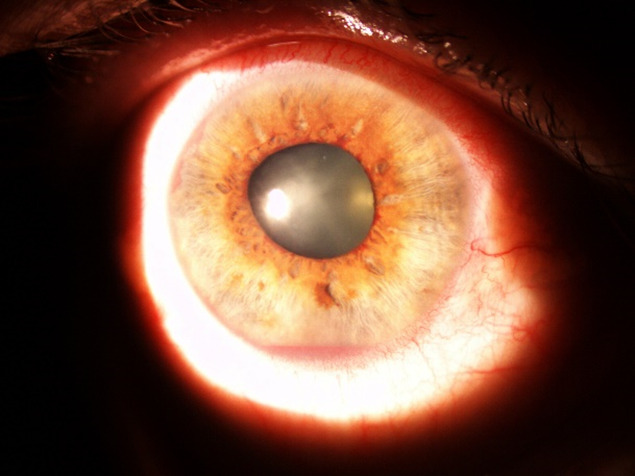
Iris neovessels with hyphema

**Fig. 2:**
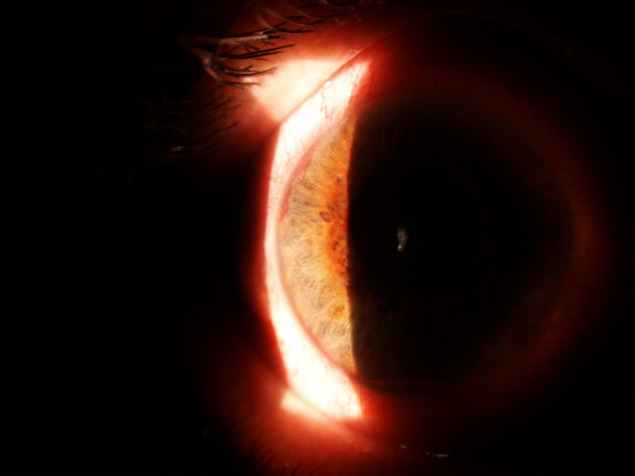
Iris neovessels

**Fig. 3:**
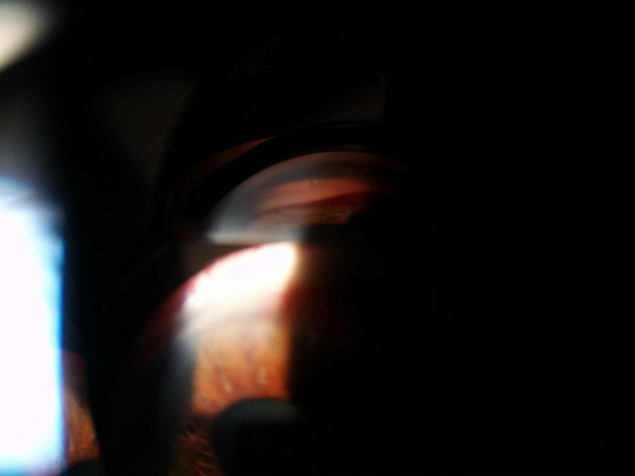
Gonioscopy image - neovessels

At the evaluation of the ocular structures, it was observed that 81,95% had conjunctival hyperemia, 68% had epithelial and stromal edema. The pupil was in fixed semi-mydriasis in 34% of cases. Iris neovessels were seen in 94% of cases (**Fig. 1**-**3**). The value of the intraocular pressure was higher than 45 mmHg in 43% of cases, in 15% of cases the intraocular pressure was between 34-43 mmHg, 8% between 24-33 mmHg and 6% lower than 24 mmHg.

**Fig. 4:**
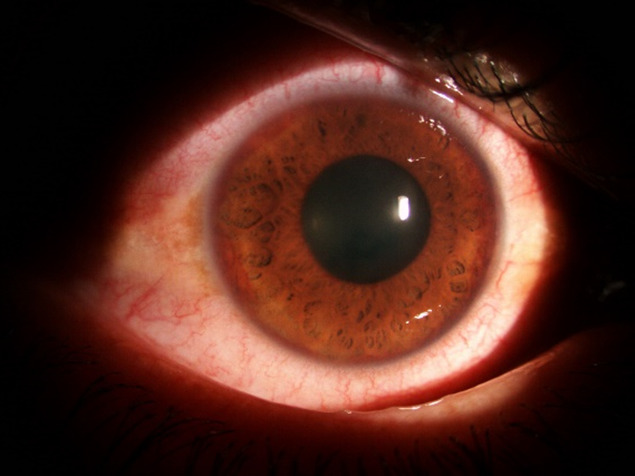
Clinical aspect after 3 weeks of intravitreal injection with Avastin

**Fig. 5:**
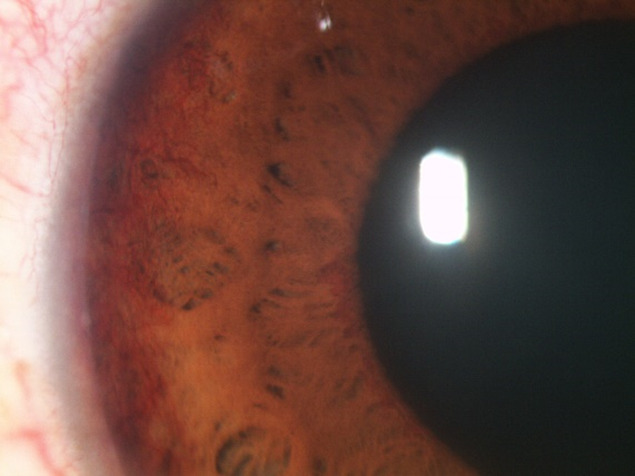
Clinical aspect after 3 weeks of intravitreal injection with Avastin

All the patients received the same treatment: local treatment with anti-glaucoma eyedrops, systemic treatment with carbonic anhydrase inhibitors (acetazolamide), Aspacardin and anti-inflammatory treatment, combined with intravitreal injections of Avastin and laser pan photocoagulation at 3-6 weeks after Avastin (**Fig. 4**,**5**).

Intravitreal injection with Avastin-technique: the skin and the eyeball are cleaned with a povidone-iodine solution, a sterile drape is applied, along with an eyelid speculum. For the next step, a local anesthesia is performed. Using the measuring caliper, a 4 mm distance is measured from the limbus or 3.5 mm in cases of patients with pseudophakia. A needle is inserted perpendicular to the sclera, and 0,2-0,5 ml of Avastin are injected intravitreal. A topical antibiotic is applied locally. In 87.5% of the eyes that received treatment with Avastin, a regression of the neovessels was seen in 4-7 days after injection. After the initiation of pan photocoagulation, the neovessels disappeared in 63.88% of the eyes. After two months, the intraocular pressure was within normal limits in 69.44% of cases. After three months, the intraocular pressure was within normal limits in 58.44% of cases. However, despite the correct treatment, two of the patients underwent evisceration of the eyeball.

## Discussions

Neovascular glaucoma represents one of the most severe complications in patients with diabetic retinopathy and center retinal vein occlusion and remains one of the leading causes of vision loss.

According to this study, Bevacizumab appears to have very good results, regardless of disease severity. A study by Mordenti et al. [**10**] shows that the half-life of Bevacizumab in the vitreous is longer than that of other molecules, due to its small size and significant diffusion through the retinal layers.

In this study, the regression of neovessels was observed in the first 4-7 days after the injection with Avastin, and maintenance of intraocular pressure within normal limits in about 60% of cases, 3 months after injection.

Better results were obtained after the initiation of pan photocoagulation, the neovessels disappearing in 63.88% of cases. 

Spitzer et al. [**11**] demonstrated in a comparative study that Bevacizumab, Pegaptanib and Ranibizumab have antiproliferative and cytotoxic effects alike.

In some cases, the poor control of high intraocular pressure leads to blindness and the eye may be very painful. In this case, the last treatment line represents the evisceration of the eyeball [**12**]. In this study, despite the correct treatment, no improvement was obtained, and two of the patients underwent evisceration of the eyeball.

## Conclusions

The etiology of secondary neovascular glaucoma is numerous, but the main cause is represented by diabetes, complicated with proliferative diabetic retinopathy, but also central retinal vein occlusion.

In our study, the main cause of neovascular glaucoma was diabetic retinopathy, a complication of diabetes mellitus. The high rate of diabetic retinopathy due to lack of glycemic control is the leading cause of vision loss. 

Most patients who are referred to a specialist have an advanced stage of the disease, without any treatment prior to presentation.

The treatment of the background disease aims to stop the evolution of neovascularization, minimize the impact on the quality of life, functional preservation of vision and preservation of the eyeball.

Given the patient’s status and the value of intraocular pressure at presentation, the combination between intravitreal injection of Avastin and retinal laser pan photocoagulation, showed good results in over 50% of the examined patients.

Prophylaxis of the background disease is the only effective method to prevent the occurrence of neovascular glaucoma. 

**Conflict of Interest statement**

The authors state no conflict of interest.

**Informed Consent and Human and Animal Rights statement**

Informed consent has been obtained from all individuals included in this study.

**Authorization for the use of human subjects**

Ethical approval: The research related to human use complies with all the relevant national regulations, institutional policies, is in accordance with the tenets of the Helsinki Declaration, and has been approved by the review board of “Carol Davila” University of Medicine and Pharmacy, Bucharest, Romania.

**Acknowledgements**

None.

**Sources of Funding**

None.

**Disclosures**

None.
